# Protein Kinase A (PKA) as a Master Regulator of the Early Metabolic Reprogramming in Bone Metastatic Prostate Cancer Cells

**DOI:** 10.1200/GO.22.35000

**Published:** 2022-05-05

**Authors:** Pablo Sanchis, Nicolas Anselmino, Rosario Lavignolle, Agustina Sabater, Estefania Labanca, Juan Bizzotto, Sofia Lage-Vickers, Gaston Pascual, Rocio Seniuk, Ayelen Toro, Nora Navone, Javier Cotignola, Elba Vazquez, Geraldine Gueron

**Affiliations:** ^1^ University of Buenos Aires, Buenos Aires, Argentina; ^2^ MD Anderson Cancer Center, Houston, TX

## Abstract

The arrival of metastatic prostate cancer (PCa) tumor cells to the bone niche requires a metabolic adaptation. We sought to identify metabolic dysregulations fueling PCa metastasis, modulated by bone secreted factors.

**METHODS:**

By an indirect co-culture system of PCa (PC3) and bone progenitors (MC3T3, pre-osteoblasts, or Raw 264.7, pre-osteoclasts) we assessed the transcriptome of PC3 cells modulated by soluble factors released from bone precursors. We validated the transcriptional profile of metabolic genes in open-access transcriptomic datasets. We performed an Ingenuity Pathway Analysis (IPA) to delineate the regulators of these metabolic genes. Bone secretome was profiled on the conditioned media (CM) by ESI-MS/MS.

**RESULTS:**

PC3 cells co-cultured with bone progenitors displayed an activation of lipidic categories, including PPAR-signaling and fat absorption/digestion. Principal Component and Unsupervised Clustering analyses using transcriptomic data from human PCa and bone metastatic samples (GSE74685) showed that the metabolic genes deregulated in PC3 accurately clustered samples in primary tumor or bone metastasis. Moreover, four lipid-associated genes, PPARA, VDR, SLC16A1 and GPX1, were associated with a shorter survival time (SU2C-PCF dataset), and were independent risk-predictors of death (*P* < .05). An IPA revealed that these genes are regulated by the Protein Kinase A (PKA). Accordingly, PC3 cells treated with the CM of the co-culture presented a decreased ATP content compared to the treatment with the CM of PC3 grown alone, which was restored upon PKA inhibition. Finally, the secretome analysis revealed soluble factors secreted by bone progenitors (Col1a1, Fn1) which could regulate PKA activity.

**CONCLUSION:**

We identified a novel lipid-associated gene signature important for metastatic PCa, triggered by the dialogue with bone cells. Moreover, PKA could regulate this signature in response to bone-secreted factors reprogramming the metabolic phenotype of metastatic cells, emerging as a potential druggable target for this disease.

